# Correction: *In Vivo* Expression Technology Identifies a Novel Virulence Factor Critical for *Borrelia burgdorferi* Persistence in Mice

**DOI:** 10.1371/journal.ppat.1004260

**Published:** 2014-06-20

**Authors:** The PLOS Pathogens Staff

Through a series of follow up experiments, the authors have come to understand that the genotype and therefore the *in vivo* phenotype of the Δ*bbk46*/vector *B. burgdorferi* clone (1470), the data for which are shown in Table 4, were incorrect. In the publication, the mutant clone was reported to contain all of the *B. burgdorferi* plasmids of the wild type parent clone; however, this finding was interpreted in error and the authors now understand that in addition to lacking gene *bbk46*, the mutant clone also lacks plasmid lp28-1. It has been reported previously that *B. burgdorferi* clones lacking lp28-1 are unable to evade the host immune response resulting in the inability of the spirochetes to persist in mice [1–5]. Because the Δ*bbk46*/vector *B. burgdorferi* clone (1470) lacks lp28-1, this suggests that the persistence phenotype (lack of spirochete reisolates in the tissues of infected mice 3 weeks post inoculation) shown in Table 4 is a result of the missing lp28-1 plasmid rather than the *bbk46* gene. The authors have just completed careful re-derivation of the Δ*bbk46*/vector *B. burgdorferi* clone (1607), genotype analysis and analysis of the phenotype of the new clone in mouse infectivity. The authors now find that the new Δ*bbk46*/vector *B. burgdorferi* clone (1607), which contains all of the *B. burgdorferi* plasmids of the wild-type parent clone, demonstrates wild type serology and tissue reisolation in the 9 out of 9 mice inoculated with 1×10^4^ spirochetes. These data confirm that the persistence phenotype reported for the Δ*bbk46*/vector *B. burgdorferi* clone (1470) in Table 4 of the publication is due to the loss of the lp28-1 plasmid and not the deletion of the *bbk46* gene. The authors have confirmed that the reported genotypes of all other clones in the publication are correct.

The conclusion that the *bbk46* gene is critical for the ability of the spirochete to evade the humoral immune response and persistently infect mice is incorrect. Therefore, all text in the Abstract, Author Summary, Introduction, Results, and Discussion relating to the infectivity phenotype of the original *bbk46* deletion mutant is invalid. Furthermore, as the *in vivo*-expressed *bbk46* gene identified in the IVET screen was incorrectly reported to be a novel virulence factor critical for *Borrelia burgdorferi* persistence in mice the corrected title for this publication should read “*In Vivo* Expression Technology Identifies Novel *Borrelia burgdorferi* Genes Expressed during an Active Murine Infection.”

The conclusions of this publication which remain valid are the development and application of *in vivo* expression technology (IVET) in *B. burgdorferi* to identify *B. burgdorferi* genes that are expressed during an active murine infection. Specifically, 289 non-identical *B. burgdorferi in vivo*-expressed sequences were identified that mapped to distinct classes of putative regulatory sequences across the genome including 80 sequences that mapped to canonical promoter positions upstream of annotated open reading frames. Characterization of the BbIVET identified candidate *in vivo*-expressed promoter sequence, *Bbive162*, which mapped immediately upstream of the uncharacterized BBK46 open reading frame on virulence plasmid lp36, revealed more than 100-fold induction during mammalian infection compared to *in vitro* growth. Furthermore, *bbk46* expression was demonstrated to be RpoS-independent. Finally, the *bbk46* open reading frame was found to be unable to produce detectable amounts of protein during *in vitro* growth and recombinant BBK46 protein was non-immunoreactive with immune serum from mice infected with *B. burgdorferi*.

In addition, the ORF associated with *Bbive* clone 290 in Table 3 should read BB0775 rather than BB0755 as originally listed and BB0181 should be noted as a homolog of FlgK.

Please see a corrected version of the article below.

1. Labandeira-Rey M, Seshu J, Skare JT (2003) The absence of linear plasmid 25 or 28-1 of *Borrelia burgdorferi* dramatically alters the kinetics of experimental infection via distinct mechanisms. Infect Immun 71: 4608–4613.
2. Labandeira-Rey M, Skare JT (2001) Decreased infectivity in *Borrelia burgdorferi* strain B31 is associated with loss of linear plasmid 25 or 28–1. Infect Immun 69: 446–455.
3. Lawrenz MB, Wooten RM, Norris SJ (2004) Effects of *vlsE* complementation on the infectivity of *Borrelia burgdorferi* lacking the linear plasmid lp28-1. Infect Immun 72: 6577–6585.
4. Purser JE, Norris SJ (2000) Correlation between plasmid content and infectivity in *Borrelia burgdorferi*. Proc Natl Acad Sci U S A 97: 13865–13870.
5. Zhang JR, Hardham JM, Barbour AG, Norris SJ (1997) Antigenic variation in Lyme disease borreliae by promiscuous recombination of VMP-like sequence cassettes. Cell 89: 275–285.

## *In Vivo* Expression Technology Identifies Novel *Borrelia burgdorferi* Genes Expressed during an Active Murine Infection

Tisha Choudhury Ellis^1^, Sunny Jain^1^, Angelika K. Linowski^1^, Kelli Rike^1^, Aaron Bestor^2^, Patricia A. Rosa^2^, Micah Halpern^1^, Stephanie Kurhanewicz^1^ and Mollie W. Jewett^1*^

^1^Burnett School of Biomedical Sciences, University of Central Florida College of Medicine, Orlando, Florida, United States of America

^2^Laboratory of Zoonotic Pathogens, Rocky Mountain Laboratories, National Institute of Allergy and Infectious Diseases, National Institutes of Health, Hamilton, Montana, United States of America

*Mollie.Jewett@ucf.edu

## Abstract

Analysis of the transcriptome of *Borrelia burgdorferi*, the causative agent of Lyme disease, during infection has proven difficult due to the low spirochete loads in the mammalian tissues. To overcome this challenge, we have developed an *In Vivo* Expression Technology (IVET) system for identification of *B. burgdorferi* genes expressed during an active murine infection. Spirochetes lacking linear plasmid (lp) 25 are non-infectious yet highly transformable. Mouse infection can be restored to these spirochetes by expression of the essential lp25-encoded *pncA* gene alone. Therefore, this IVET-based approach selects for *in vivo-*expressed promoters that drive expression of *pncA* resulting in the recovery of infectious spirochetes lacking lp25 following a three week infection in mice. Screening of approximately 15,000 clones in mice identified 289 unique *in vivo*-expressed DNA fragments from across all 22 replicons of the *B. burgdorferi* B31 genome. The *in vivo*-expressed candidate genes putatively encode proteins in various functional categories including antigenicity, metabolism, motility, nutrient transport and unknown functions. Candidate gene *bbk46* on essential virulence plasmid lp36 was found to be highly induced *in vivo* and to be RpoS-independent. The *bbk46* gene was dispensable for *B. burgdorferi* infection in mice. Our findings highlight the power of the IVET-based approach for identification of *B. burgdorferi in vivo*-expressed genes, which might not be discovered using other genome-wide gene expression methods. Further investigation of the novel *in vivo*-expressed candidate genes will contribute to advancing the understanding of molecular mechanisms of *B. burgdorferi* survival and pathogenicity in the mammalian host.

## Author summary

Lyme disease is caused by tick-bite transmission of the pathogenic spirochete *Borrelia burgdorferi*. An increased understanding of how *B. burgdorferi* survives throughout its infectious cycle is critical for the development of innovative diagnostic and therapeutic protocols to reduce the incidence of Lyme disease. One of the major difficulties blocking this effort has been genome-wide identification of the *B. burgdorferi* genes that are expressed in the mammalian host environment. Using *in vivo* expression technology (IVET) in *B. burgdorferi* for the first time, we have identified *B. burgdorferi* genes that are expressed during an active murine infection. We demonstrate that candidate gene *bbk46*, encoded on essential linear plasmid 36, is highly expressed *in vivo* and, unlike some other known *B. burgdorferi in vivo*-induced genes, is not RpoS regulated. The *bbk46* gene, however, was not found to be required for *B. burgdorferi* infection in mice. Further studies focused on analysis of the novel *B. burgdorferi in vivo*-expressed genes identified using IVET will provide insight into the ability of this pathogen to survive in the mammalian host.

## Introduction

Lyme disease is a multi-stage inflammatory disease caused by the pathogenic spirochete *Borrelia burgdorferi*, which is transmitted by the bite of an infected tick [1]. *B. burgdorferi* has an enzootic life cycle that requires persistence in two disparate environments, the arthropod vector and the mammalian host. *B. burgdorferi* is well adapted to modulate its expression profile in response to the different conditions encountered throughout its infectious cycle [2]. Although the specific environmental signals that induce changes in spirochete gene expression are not fully defined, it has been reported that changes in temperature, pH, the presence or absence of mammalian blood, as well as changes in bacterial growth rate, can affect patterns of gene expression [2–8]. DNA microarray analysis and proteomics have been used to examine changes in the global expression profile of *B. burgdorferi* grown under *in vitro* conditions that partially mimic the tick and mouse environments [3–5]. A rat dialysis membrane chamber (DMC) implant model, together with microarray technology, has been used to help identify *B. burgdorferi* genes expressed in response to mammalian host-specific signals [7–10]. Although the data reported in these studies provide insight into the molecular mechanisms of gene regulation, they may not fully reflect the patterns of *B. burgdorferi* gene expression during an active mammalian infection. Furthermore, transcriptome analysis of *B. burgdorferi* during murine infection has proven difficult given that spirochete loads in the blood and tissues are too low to recover sufficient spirochete RNA for direct microarray analysis [11].

*In vivo* expression technology (IVET) is a gene discovery method used to identify transcriptionally active portions of a microbial genome during interaction of the microorganism with a particular environment or host organism [12,13]. In this system, the environment itself directly selects for upregulated bacterial loci [14]. The IVET selection system functions on the premise that deletion of a biosynthetic gene can lead to attenuation of growth and persistence of a pathogen in the host environment. This attenuation can be complemented by expression of the biosynthetic gene driven by promoters that are transcriptionally active *in vivo*. Thus, in the environment of interest, *in vivo* transcriptionally active promoters can be selected from a genomic library of DNA fragments cloned upstream of the essential biosynthetic gene [12–15]. IVET is a sensitive and versatile method for identification of *in vivo*-expressed genes that has been used with pathogenic bacteria and fungi in a wide variety of host environments and has identified a number of previously uncharacterized virulence genes [14–18].

Using IVET we have developed and applied a genome-wide genetic screening approach to identify *B. burgdorferi* genes that are expressed during an active murine infection. This is the first time that an IVET strategy has been applied to *B. burgdorferi* Furthermore, this approach resulted in the identification of novel *B. burgdorferi in vivo*-expressed genes.

## Results

**Mammalian host-adapted spirochetes demonstrate a 100-fold decrease in ID_50_ relative to *in vitro* grown spirochetes.** It is clear that *B. burgdorferi* modulates its gene expression profile at different stages of the infectious cycle [2]. The infectious dose of wild-type *B. burgdorferi* varies depending on the environment from which the spirochetes are derived. For example, the 50% infectious dose (ID_50_) of spirochetes derived from partially fed ticks has been found to be two orders of magnitude lower than that of log phase *in vitro* grown *B. burgdorferi* [19]. In order to quantitatively assess the impact that adaptation to the mammalian environment has on *B. burgdorferi* infectivity the 50% infectious dose (ID_50_) of spirochetes derived directly from the mammalian host was determined and compared to that of log phase *in vitro* grown spirochetes. *B. burgdorferi* are only transiently present in the blood of immunocompetent mice [20], whereas spirochetes persist longer in the blood of immunocompromised mice [21]. Therefore, the blood of severe combined immunodeficiency (*scid*) mice infected with *B. burgdorferi* was used as a source of spirochetes adapted to the mammalian environment. Strikingly, an inoculum containing approximately eight *in vivo*-derived spirochetes was able to infect five out of six mice, whereas, 5,000 *in vitro* grown spirochetes were required to obtain this level of infectivity (Table 1). The ID_50_ for *in vivo*-derived spirochetes was found to be less than eight organisms. In contrast, the ID_50_ for *in vitro* grown spirochetes was calculated to be 660 organisms. These data indicate that mammalian host-adapted spirochetes are 100-fold more infectious than *in vitro* grown spirochetes, likely due to appropriate coordinate expression of *in vivo*-expressed genes important for murine infectivity.

**Table 1:** *In vivo*-adapted *B. burgdorferi* are highly infectious.

*In vivo* grown spirochetes		*In vitro* grown spirochetes	
Spirochete dose	Number of mice infected^a^/number of mice analyzed	Spirochete dose	Number of mice infected^a^/number of mice analyzed
8×10^2^	6/6	5×10^4^	6/6
8×10^1^	6/6	5×10^3^	6/6
8×10^0^	5/6	5×10^2^	2/6
		5×10^1^	1/6
		5×10^0^	0/6
ID_50_ b	<8 spirochetes	ID_50_ b	660 spirochetes

a Mouse infection was determined 3 weeks post inoculation by serological response to *B. burgdorferi* proteins and reisolation of spirochetes from ear, bladder and joint tissues.

b The ID_50_ was calculated according to method of Reed and Muench [83].

**The *B. burgdorferi* IVET system is a robust method for selection of *B. burgdorferi* sequences that are expressed during murine infection.** We have developed a genome-wide genetic screening method to identify *B. burgdorferi* genes that are expressed during mouse infection using an *in vivo* expression technology (IVET) approach [12,13]. The *in vivo* expression technology vector, pBbIVET, carries the *B. burgdorferi bmpB* Rho-independent transcription terminator sequence [22] repeated in triplicate (3XTT), to prevent any read-through promoter activity from the pBSV2* *Borrelia* shuttle vector backbone, followed by the promoter-less *in vivo*-essential *pncA* gene (Figure 1) [23,24]. Spirochetes lacking linear plasmid (lp) 25 are non-infectious in mice and severely compromised in the tick vector [25–31]. The *pncA* gene, located on lp25, encodes a nicotinamidase that is sufficient to restore murine infectivity to *B. burgdorferi* lacking the entire lp25 plasmid [24]. Genetic transformation of low-passage, infectious *B. burgdorferi* occurs at low frequency and efficiency hampering introduction of a complex DNA library into an infectious background [32,33]. Because *B. burgdorferi* clones lacking lp25 and lp56 demonstrate increased transformability [34], we isolated a clonal derivative of the low-passage infectious clone A3 that lacks both lp25 and lp56. This clone was designated A3 68-1 [35]. Clones A3 and A3 68-1 were transformed by electroporation with 20 µg of a *Borrelia* shuttle vector. The transformation frequency and efficiency of A3 68-1 was determined relative to that of the A3 parent. As expected, genetic transformation of A3 68-1 occurred at a high frequency and efficiency. We recovered approximately 2,000 transformants/ml in clone A3 68-1, whereas no transformants were recovered with a parallel transformation of clone A3 (data not shown). In order to test the function of the *B. burgdorferi* IVET system, the promoter for the *in vivo* essential *ospC* gene [36], was cloned in front of the promoter-less *pncA* gene in pBbIVET (Figure 1), creating plasmid pBbIVET-*ospC*_p_. This plasmid, along with pBbIVET alone, was transformed into the non-infectious, low-passage, highly transformable *B. burgdorferi* clone A3 68-1. All clones were tested for their abilities to infect groups of 6 C3H/HeN mice at an infectious dose 100 (ID_100_) of 1×10^4^ spirochetes [37], indicating the presence or absence of an active promoter sufficient to drive expression of *pncA* thereby restoring infectivity. Spirochetes were reisolated from the ear, bladder and joint tissues of 5/6 mice infected with *B. burgdorferi* harboring pBbIVET-*ospC*_p_. No spirochetes were reisolated from mice (0/6) infected with *B. burgdorferi* carrying the promoter-less pBbIVET alone. Together these data demonstrated that our promoter trap system functioned with a known *in vivo* active promoter.

**Figure 1:**
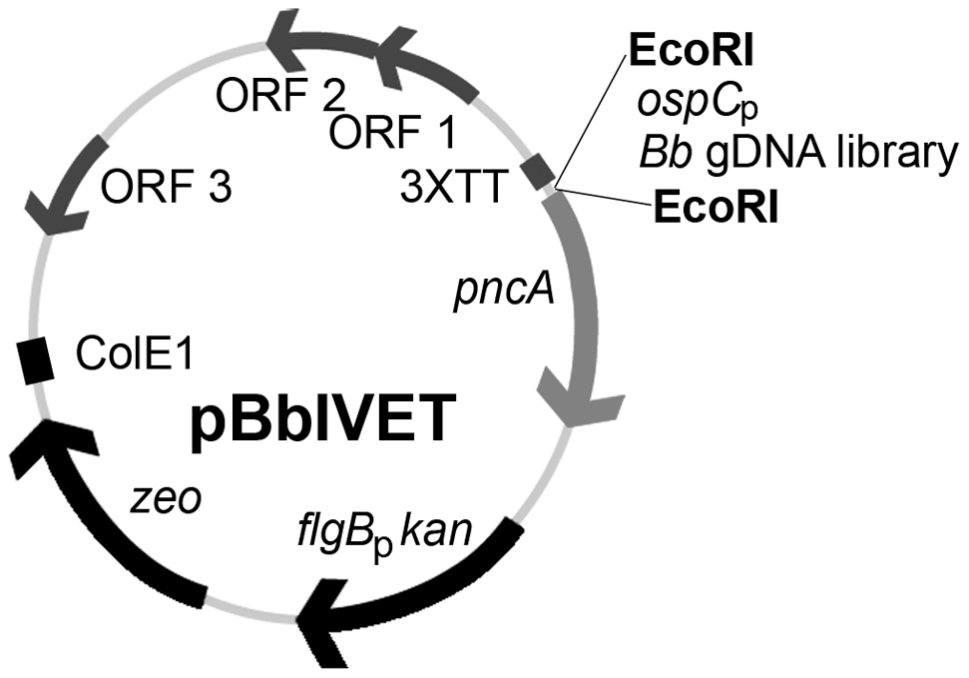
Schematic representation of the pBbIVET vector. Features of this vector include: 3XTT, the transcriptional terminator sequence for *bmpB* [22] repeated in triplicate; *pncA*, promoterless *pncA* gene; *flgB*_p_ *kan*, kanamycin resistance cassette; *zeo*, zeocin resistance marker; ColE1, *E. coli* origin of replication; ORFs 1, 2, 3, *B. burgdorferi* cp9 replication machinery. The EcoRI restriction site was used to clone the *B. burgdorferi* control *in vivo*-expressed promoter, *ospC*_p_, as well as the *B. burgdorferi* (*Bb*) gDNA library, in front of the promoterless *pncA* gene. The pBbIVET vector was derived from the *B. burgdorferi* shuttle vector pBSV2* [80].

**Screening for *B. burgdorferi* genes expressed during murine infection.** A *B. burgdorferi* genomic DNA library using an average DNA fragment size of approximately 200 bps was constructed upstream of the promoter-less *pncA* gene (Figure 1) in the pBbIVET vector in *E. coli*, yielding approximately 30,000 independent clones. A small subset of individuals from the 30,000 clone library in *E. coli* was analyzed by PCR and DNA sequencing and determined to carry non-identical *B. burgdorferi* DNA fragments. The strategy used to construct the pBbIVET library allowed the DNA fragments to be cloned in either the forward or reverse orientation relative to the *pncA* gene. Therefore, a library of 30,000 clones each harboring a unique 200 bp DNA fragment represented approximately 2X coverage of the 1.5 Mb genome of *B. burgdorferi*. Although the initial analysis of the transformation efficiency of *B. burgdorferi* clone A3 68-1 demonstrated that each transformation of 20 µg of a single purified plasmid into this genetic background yielded approximately 10,000 transformants, this transformation efficiency was not achieved when 20 µg of complex library plasmid DNA was transformed into A3 68-1. Forty four transformations of the library plasmid DNA resulted in recovery of approximately 15,000 individual clones in *B. burgdorferi* A3 68-1, representing an IVET library in *B. burgdorferi* with approximately 1X coverage of the spirochete genome. As described for the pBbIVET library in *E. coli*, a subset of individuals from 15,000 clone library in *B. burgdorferi* were analyzed and found to carry non-identical *B. burgdorferi* DNA fragments.

Like the BbIVET system described here, many IVET strategies are based upon complementation of auxotrophy. For microorganisms other than *B. burgdorferi* these strategies have allowed negative selection against “promoter-less” clones in minimal medium in which the auxotroph mutants are unable to grow [38]. *B. burgdorferi* lacking *pncA* are not attenuated for growth in the complex, undefined *B. burgdorferi* medium, BSKII. Moreover, there is currently no minimal medium available that supports the growth of wild-type *B. burgdorferi*. Therefore, the BbIVET system did not include negative selection against “promoter-less” clones *in vitro*. 179 mice were infected with pools from the *B. burgdorferi* IVET library of approximately 100 clones each, with each clone at a dose of 1×10^4^ spirochetes resulting in a total dose of 1×10^6^ spirochetes per mouse. Three weeks post inoculation, mice were sacrificed and ear, heart, bladder and joint tissues were harvested for reisolation of infectious spirochetes. 175 out of 179 mice became infected with *B. burgdorferi* as determined by reisolation of spirochetes from at least two or more of the tissue sites analyzed (Table 2). However, due to the potentially stochastic nature of the kinetics of infection [39] and/or tissue-specific promoter activity of distinct *B. burgdorferi* genomic fragments not all four tissue sites from all 175 infected mice were found to be positive for spirochete reisolation. Nonetheless, the recovery of live spirochetes from infected mouse tissues suggested that these spirochetes harbored *in vivo* active promoter(s) in the pBbIVET plasmid sufficient to drive expression of the *in vivo*-essential *pncA* gene to restore spirochete mouse infectivity. Total genomic DNA was isolated from each pool of reisolated spirochetes from each of the four mouse tissues and the pBbIVET plasmid DNA rescued in *E. coli*. Colony PCR using primers targeting the genomic DNA insert region of the pBbIVET vector was performed on 24 of the resulting *E. coli* colonies from each plasmid rescue transformation. No reisolated spirochetes were found to harbor a pBbIVET plasmid lacking a genomic DNA fragment insert. The amplified inserts were analyzed by restriction digest using a cocktail of the A/T-rich restriction enzymes to identify those DNA fragments with distinct restriction patterns, suggesting that these fragments represent different *in vivo* active promoters. Up to eleven non-identical restriction digest patterns were detected for every subset of 24 *E. coli* transformants carrying pBbIVET DNA that were analyzed (Figure S1). The DNA fragments corresponding to each distinct restriction digest pattern were further analyzed by DNA sequencing and the identities of the sequences determined by microbial genome BLAST analysis. Screening of approximately 15,000 BbIVET clones through mice resulted in the identification of 289 non-identical *B. burgdorferi in vivo*-expressed (*Bbive*) DNA fragments from across the chromosome and all 21 plasmid replicons of the *B. burgdorferi* B31 segmented genome (Table 2). Although the 1∶1 molar ratio of insert to vector used to generate the pBbIVET library did not preclude insertion of more than one fragment into each clone, only 20 out of the 289 clones were found to harbor two distinct DNA fragments. Of these clones the 3′ DNA fragment, proximal to the *pncA* ORF, was assumed to be the active promoter and was included in the subsequent analyses.

**Table 2:** The *B. burgdorferi* IVET system selects for *in vivo*-active promoters.

Number of BbIVET clones screened	Positive reisolation of infectious spirochetes from mouse tissues^a^				Number of unique genomic fragments recovered
	Ear	Heart	Bladder	Joint	
∼15,000	175/179	173/179	172/179	174/179	289

a Number of mice positive for spirochete reisolation/ number of mice analyzed. Four mice were reisolate-negative for all tissues analyzed. Three mice were reisolate-negative for the bladder tissue. One mouse was reisolate-negative for the heart and joint tissues. One mouse was reisolate-negative for the heart tissue.

***B. burgdorferi in vivo* expressed promoters map to distinct classes of putative regulatory sequences across the genome.** Genomic mapping of the 289 unique *Bbive* promoters identified in this genetic screen demonstrated that 67% of the sequences mapped to sense DNA in the same direction as annotated open reading frames, 27% mapped to antisense DNA in the opposite direction to annotated open reading frames and 6% mapped to intergenic regions lacking annotated open reading frames. Of the large percentage of sense sequences, 41%, which represented 28% of the total *Bbive* sequences, mapped to regions just upstream of and in the same orientation to annotated open reading frames, suggesting that these sequences are promoters for the associated open reading frames and that these open reading frames are candidate *in vivo*-expressed genes. The remaining 59% of the sense sequences, which represented 39% of the total *Bbive* sequences, mapped within annotated open reading frames, suggesting the possibility for promoter elements within *B. burgdorferi* genes. Similar findings of putative transcriptional start sites within genes and operons have been reported for other bacterial pathogens [40,41]. The sequences that mapped to putative promoter locations in the genome and the genes associated with these promoters were prioritized for further analysis. Among the list of 80 sequences, 9 promoter regions were represented by two overlapping genomic DNA fragments. Five of these overlapping sequence pairs shared the same 3′ end, suggesting that the sequences belonging to each pair contained the same promoter. Whereas, the other four overlapping sequence pairs harbored distinct 3′ ends, suggesting that each sequence contained a unique promoter. The 71 *in vivo*-expressed candidate genes have been annotated to encode proteins in various functional categories including: cell division, cell envelope, replication, metabolism, motility, protein synthesis, transport and unknown functions (Table 3).

**Table 3:** *B. burgdorferi in vivo*-expressed candidate genes organized by functional category.

*Bbive* clone^a^	Replicon	ORF^b^	Protein designation, Annotated function^c^
**Cell division**			
289	chromosome	BB0715	FtsA cell division protein
**Cell envelope**			
15	chromosome	BB0213	Putative lipoprotein
94	chromosome	BB0760	Gp37 protein
175	lp54	BBA36	Lipoprotein
271	lp54	BBA57	Lipoprotein
297	lp25	BBE16	BptA
151	lp28-2	BBG01	Putative lipoprotein
267	lp38	BBJ34	Putative lipoprotein
269	lp38	BBJ51	VlsE paralog, pseudogene
162	lp36	BBK46	Immunogenic protein P37, authentic frameshift
77	cp32-8, cp32-3, cp32-7, cp32-9, lp56, cp32-4,cp32-6, cp32-1	BBL28, BBS30, BBO28, BBN28, BBQ35, BBR28, BBM28, BBP28	Mlp lipoprotein family
**DNA replication**			
274	chromosome	BB0111	DnaB replicative helicase
226	chromosome	BB0632	RecD exodeoxyribonuclease V, alpha chain
152	lp28-3	BBH13	RepU replication machinery
**Energy metabolism**			
62	chromosome	BB0057	Gap glyceraldehyde-3-phosphate dehydrogenase, type 1
34	chromosome	BB0327	Glycerol-3-phosphate O acyltransferase
44	chromosome	BB0368	NAD(P)H-dependent glycerol-3-phosphate dehydrogenase
47	chromosome	BB0381	Trehalase
81	chromosome	BB0676	Phosphoglycolate phosphate
**Fatty acid and phospholipid metabolism**			
85	chromosome	BB0704	AcpP acyl carrier protein
**Motility and chemotaxis**			
14	chromosome	BB0181	FlgK flagellar hook-associated protein
29	chromosome	BB0293	FlgB flagellar basal body rod
290	chromosome	BB0775	Flagellar hook-basal body complex protein
65	chromosome	BB0551	CheY-1 chemotaxis response regulator
222	chromosome	BB0568	Chemotaxis response regulator protein-glutamate methylesterase
**Prophage function**			
295	cp32-8, cp32-7, cp32-1, cp32-3, cp32-6, cp32-4	BBL23, BBO23, BBP23, BBS23, BBM23, BBR23	Holin BlyA family
**Protein fate**			
193	chromosome	BB0031	LepB signal peptidase I
**Protein synthesis**			
202	chromosome	BB0113	RpsR ribosomal protein S18
216, 217	chromosome	BB0485	RplP ribosomal protein L16
58	chromosome	BB0495	RpsE 30S ribosomal protein S5
59	chromosome	BB0496	50S ribosomal protein L30
219	chromosome	BB0503	RplQ ribosomal protein L17
232	chromosome	BB0660	GTP-binding Era protein
288	chromosome	BB0682	TrmU tRNA (5-methylaminomethyl-2-thiouridylate)-methyltransferase
**Regulation**			
208	chromosome	BB0379	Protein kinase C1 inhibitor
50	chromosome	BB0420	Hk1 histidine kinase
**Nucleoside salvage**			
148	lp25	BBE07	Pfs protein, pseudogene
**Transcription**			
1	chromosome	BB0389	RpoB DNA-directed RNA polymerase, beta subunit
287	Chromosome	BB0607	PcrA ATP-dependent DNA helicase
84	chromosome	BB0697	RimM 16S rRNA processing protein
**Transport**			
204	chromosome	BB0318	MglA methylgalactoside ABC transporter ATP-binding protein
46	chromosome	BB0380	MgtE Mg^2+^ transport protein
**Unknown**			
56	chromosome	BB0049	Hypothetical protein
69	chromosome	BB0063	Pasta domain protein
2	chromosome	BB0102	Conserved hypothetical
8	chromosome	BB0138	Conserved hypothetical
13	chromosome	BB0176	ATPase family associated with various cellular activities
23	chromosome	BB0265	Conserved hypothetical
212	chromosome	BB0428	Conserved hypothetical
52, 53	chromosome	BB0429	Conserved hypothetical
220	chromosome	BB0504	Conserved hypothetical
67	chromosome	BB0562	Conserved hypothetical
223	chromosome	BB0577	Conserved hypothetical
71	chromosome	BB0592	Caax amino protease family
73	chromosome	BB0619	DHH family phosphoesterase function
96	chromosome	BB0799	Conserved hypothetical
240	cp26	BBB27	Unknown essential protein
145, 146	lp25	BBE0036	Hypothetical protein
147	lp25	BBE01	Conserved hypothetical
265, 266	lp38	BBJ30	Conserved hypothetical
171	lp38	BBJ36	Conserved hypothetical
75,173	lp38	BBJ46	Conserved hypothetical
129, 136, 296	cp32-8, cp32-1, cp32-7, lp56, cp32-9	BBL41, BBP40, BBO42, BBQ48, BBN41	Conserved hypothetical
130, 249	cp32-8, cp32-1, cp32-6	BBL42, BBP41, BBM41	Conserved hypothetical
117, 177	cp32-6, lp56, cp32-9, cp32-8, cp32-3, cp32-1, cp32-4	BBM18, BBQ25, BBN18, BBL18, BBS18,BBP18, BBR18	Conserved hypothetical
182, 183	lp56	BBQ41	PF-49 protein
188	lp56	BBQ84.1	Conserved hypothetical
189	lp56, lp28-3, lp17	BBQ89, BBH01, BBD01	Conserved domain protein
244	cp32-4, cp32-3, cp32-6, p56, cp32-8, cp32-9	BBR05, BBN05, BBM05, BBQ12, BBL05, BBO05, BBP05	Lyme disease protein of unknown function

a In some cases two or more *Bbive* clones shared overlapping, non-identical sequence, as indicated by multiple *Bbive* clone numbers.

b ORF, open reading frame that maps just downstream and in the same orientation to the *Bbive* sequence.

c Annotation described by Fraser *et al.* [45].

**Table 4:** The *bbk46* gene is dispensable for *B. burgdorferi* mouse infection by needle inoculation.

Clone	Serology^a^	Positive reisolation of spirochetes from mouse tissues^b^		
		Ear	Bladder	Joint
Δ*bbk46*/vector	9/9	9/9	9/9	9/9
Δ*bbk46*/*bbk46* ^+^	5/6	5/6	5/6	5/6

a Determined 3 weeks post inoculation by serological response to *B. burgdorferi* total protein lysate.

b Number of mice positive for spirochete reisolation/ number of mice analyzed. NA, not applicable.

**IVET identified candidate gene *bbk46* on virulence plasmid lp36.** Linear plasmid 36 is required for *B. burgdorferi* mouse infection; however, the genetic elements on lp36 that contribute to this phenotype have not been fully defined [37]. IVET identified a candidate *in vivo*-expressed promoter sequence, *Bbive162*, which mapped to lp36. This sequence was found to be 60 bp long, with 48 bp immediately upstream of and in the same direction as the BBK46 open reading frame (Figure 2), suggesting that the *bbk46* gene may be expressed during mammalian infection and may contribute to the essential role of lp36 in *B. burgdorferi* infectivity. Therefore, the *bbk46* gene was selected for further analysis.

**Figure 2:**
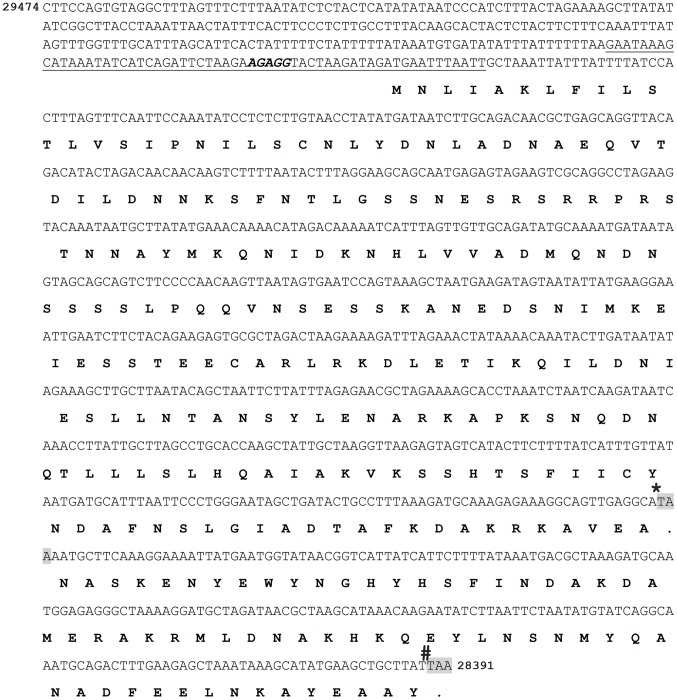
The nucleotide and putative amino acid sequence of the BBK46 open reading frame. The reverse complement of nucleotides 28391 to 29474 on lp36, encompassing *bbk46* (Genbank GeneID: 1194234) and its putative promoter sequence. The nucleotide sequence of *Bbive162* is underlined. The putative ribosome binding site is shown in bold italics. The putative BBK46 amino acid sequence is shown in bold. The stop codons at nucleotides 625 and 820 are highlighted in gray. The position of the inserted FLAG-epitope tag sequence is indicted with a star (*). The position of the inserted cMyc-epitope tag sequence is indicated with a number sign (**#**).

**Expression of the *bbk46* gene is induced during murine infection.** Our BbIVET screen identified gene *bbk46* as a putative *in vivo*-expressed gene. The BbIVET screen was designed to identify *B. burgdorferi* DNA fragments that are expressed *in vivo* and did not discriminate between those promoters that are specifically induced *in vivo* and those promoters that are expressed both *in vitro* and *in vivo*. Therefore, quantitative reverse transcription PCR (qRT-PCR) was used to validate the expression of *bbk46 in vivo* and to determine whether *bbk46* expression was upregulated *in vivo* compared to *in vitro*. Total RNA was isolated from bladder tissue collected from mice infected with 1×10^5^ wild-type *B. burgdorferi* three weeks post-inoculation as well as log phase *in vitro* grown spirochetes. RNA was converted to cDNA using random hexamer primers and the mRNA level of each target gene was measured relative to the constitutive *recA* gene using quantitative PCR. The gene expression levels of *flaB* and *ospC* were also measured as control constitutively-expressed and *in vivo*-induced genes, respectively. These data demonstrated that although *bbk46* was expressed during *in vitro* growth, expression of this gene was increased more than 100-fold during mammalian infection (Figure 3A). Consistent with their known patterns of gene regulation, *flaB* expression was relatively unchanged *in vivo* compared to *in vitro*; whereas, *ospC* demonstrated a nearly 1000-fold increase in expression *in vivo* compared to *in vitro* (Figure 3A). Moreover, the relative amount of *bbk46* expression during *in vitro* growth was found to be approximately 10-fold more than that of *ospC*. Whereas, the *in vivo* expression levels of genes *bbk46*, *ospC* and *flaB* were similar.

**Figure 3:**
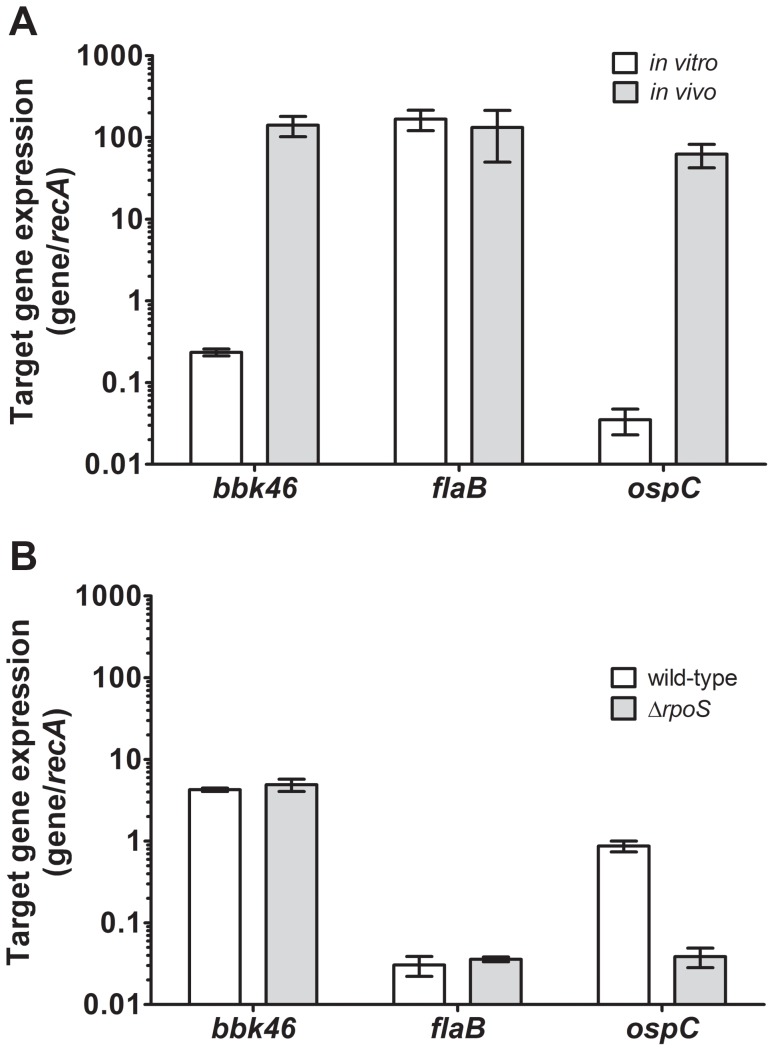
Expression of the *bbk46* gene is upregulated during murine infection and is RpoS-independent. Total RNA was isolated from bladder tissue collected from (A) mice infected with 1×10^5^ wild-type *B. burgdorferi* three weeks post-inoculation (*in vivo*, gray bars) and from log phase *in vitro* grown spirochetes (*in vitro*, white bars) or (B) stationary phase temperature-shifted stationary phase *in vitro* grown wild-type (white bars) or Δ*rpoS* (gray bars) *B. burgdorferi*. RNA was reverse transcribed to cDNA using random hexamer primers. The expression of *bbk46*, *flaB* and *ospC* were quantified using quantitative reverse transcription polymerase chain reaction (qRT-PCR) and a standard curve analysis method. The mRNA levels of the *bbk46, flaB* and *ospC* gene transcripts were normalized to that of the constitutive *recA* gene. The data are expressed as the gene transcript/*recA* transcript. The data represent the average of triplicate qRT-PCR analyses of 3 biological replicates. Error bars represent the standard deviation from the mean.

RpoS is a global regulator that controls expression of genes expressed during mammalian infection, including *ospC* [2]. Because *bbk46* expression was induced *in vivo* in a manner similar to that of *ospC*, we sought to determine whether, like *ospC*, *bbk46* is an RpoS-regulated gene. RNA was isolated from stationary phase temperature-shifted wild-type and Δ*rpoS* mutant spirochetes, a growth condition previously shown to induce expression of *rpoS* and *rpoS*-regulated genes [42]. Quantitative RT-PCR was then performed for genes *bbk46*, *flaB, ospC* and *recA*, as described above. As expected, *ospC* expression was increased approximately 20 times in the presence compared to the absence of *rpoS* (Figure 3B). In contrast, *bbk46* expression was RpoS-independent under these growth conditions (Figure 3B). Likewise, no RpoS-dependent change in gene expression was detected for *flaB*. Interestingly, the amount of *flaB* expression detected in the stationary phase temperature-shifted spirochetes (Figure 3B) was dramatically decreased compared to the amount of *flaB* expression detected in log phase and *in vivo* grown spirochetes (Figure 3A), suggesting that *flaB* is not expressed at the same level under all growth conditions. Together these data demonstrated that *bbk46* was highly induced during murine infection and *bbk46* expression was not controlled by RpoS during *in vitro* growth.

**The *bbk46* open reading frame fails to produce detectable amounts of protein during *in vitro* growth.** The *bbk46* gene is a member of paralogous gene family 75, which also includes lp36-encoded genes *bbk45*, *bbk48* and *bbk50*, all of which are annotated to encode putative P37 immunogenic lipoproteins [43–45] (Figure 4). These genes are located on the right arm of lp36 in *B. burgdorferi* clone B31, which is a highly variable region among distinct *B. burgdorferi* isolates [44]. The members of paralogous gene family 75 are conserved within *B. burgdorferi* isolates but are not present in the relapsing fever spirochetes. The *B. burgdorferi* clone B31 BBK45, BBK48 and BBK50 proteins are predicted to be 301, 288 and 332 amino acids, respectively. However, *B. burgdorferi* clone B31 *bbk46* is annotated as a pseudogene as a result of an authentic frame shift resulting in a TAA stop codon at nucleotide 625 [43–45], thereby producing a putative 209 amino acid protein. In contrast, the BBK46 homolog in clone N40, BD04, harbors a CAA codon at nucleotide 625, resulting in a glutamic acid residue at amino acid 209 and producing a putative 273 amino acid protein [46]. Sequence analysis of the cloned *bbk46* open reading frame confirmed the presence of the TAA stop codon at nucleotide 625 (Figure 2). To experimentally determine the size of the BBK46 protein produced in *B. burgdorferi* B31 the *bbk46* ORF along with a FLAG epitope tag sequence prior to the stop codon at nucleotide 625 and a cMyc epitope tag sequence prior to the stop codon at nucleotide 820 (Figure 2) was cloned into the *B. burgdorferi* shuttle vector pBSV2G under the control of either the constitutive *flaB* promoter or the putative endogenous *bbk46* promoter. A mutant clone lacking the entire BBK46 open reading frame was constructed by allelic exchange and verified by PCR analysis (Figure 5A and 5B). The *bbk46* mutant clone was transformed with the shuttle vectors carrying the epitope tagged *bbk46* constructs. BBK46 protein production was assessed in both *E. coli* and *B. burgdorferi*. Immunoblot analyses using αFLAG and αcMyc monoclonal antibodies resulted in detection of a FLAG-epitope tagged protein of an approximate molecular mass of 23 kDa, which is the predicted size of the 209 amino acid BBK46 protein, in the *E. coli* clones carrying both the *flaB*_p_-driven and the *bbk46*_p_-driven constructs (Figure 6). Surprisingly, no FLAG epitope tagged protein was detected in either *B. burgdorferi* clone (Figure 6), although *bbk46* gene expression was observed in these clones (data not shown), indicating that the lack of detectable BBK46 protein was not likely the result of a transcription defect. Furthermore, no cMyc epitope tagged protein was detected in either *E. coli* or *B. burgdorferi*. Together these data suggested that although the *bbk46* ORF is competent to produce a 23 kDa protein in *E. coli* and the transcript is expressed in *B. burgdorferi* during *in vitro* growth, the protein is either not produced or is rapidly turned over in log phase *in vitro* grown *B. burgdorferi*.

**Figure 4:**
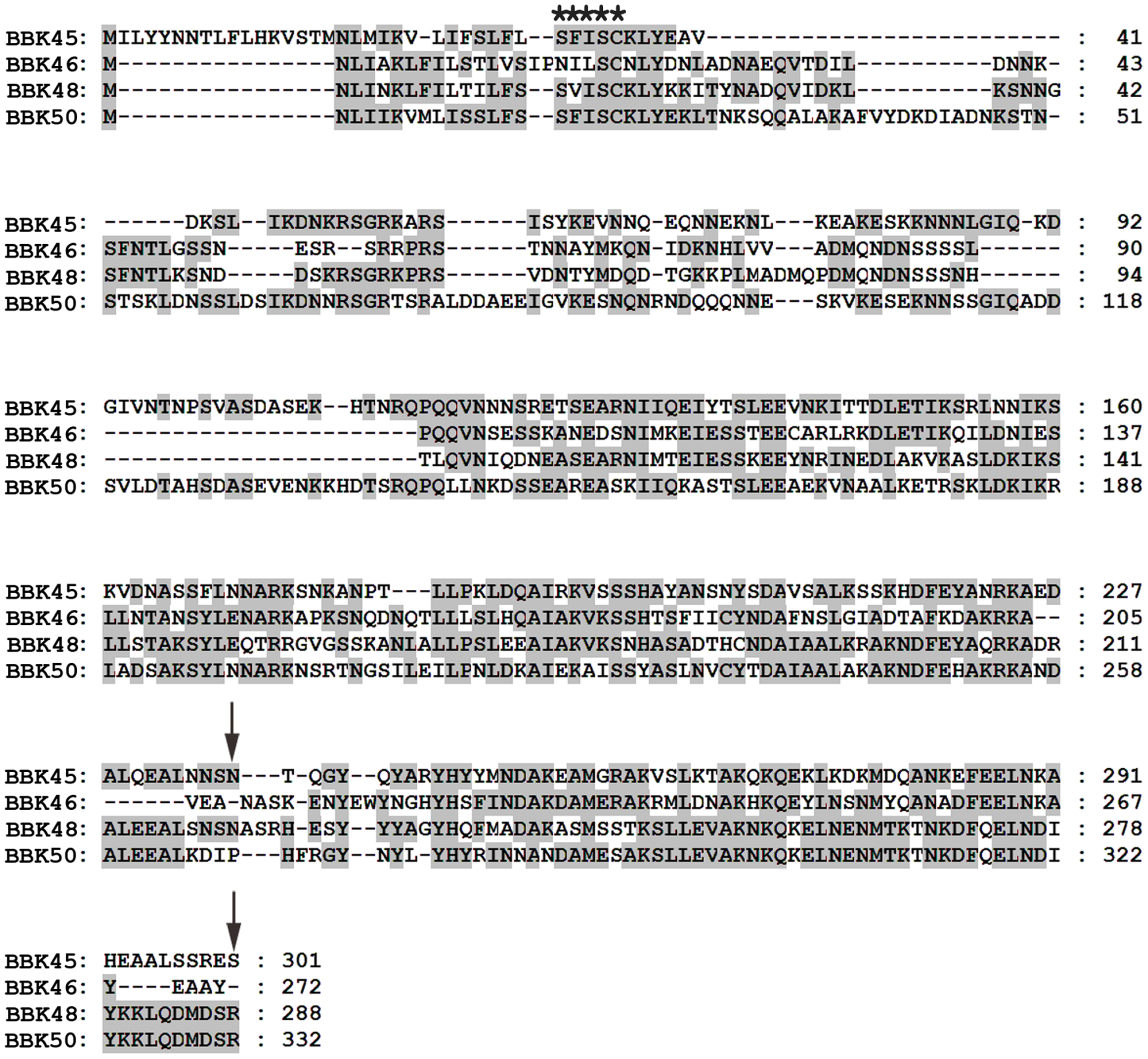
Amino acid alignment of the putative members of the immunogenic protein P37 family encoded on lp36. Shown is an amino acid alignment of the P37 protein family members BBK45 (GenBank accession no. NP_045617.2), BBK46 (translated *bbk46*, Genbank GeneID: 1194234), BBK48 (GenBank accession no. NP_045619.1) and BBK50 (GenBank accession no. NP_045621.1). Amino acids identical to the consensus sequence are shaded. The predicted SpLip lipobox sequence [81] is indicted with five stars. Dashes represent spaces introduced for optimal sequence alignment. The positions of the two stop codons in the *bbk46* translation are indicated with arrows. Amino acid sequences were aligned using the CLUSTAL W algorithm in the MEGALIGN program from the DNASTAR Lasergene suite.

**Figure 5:**
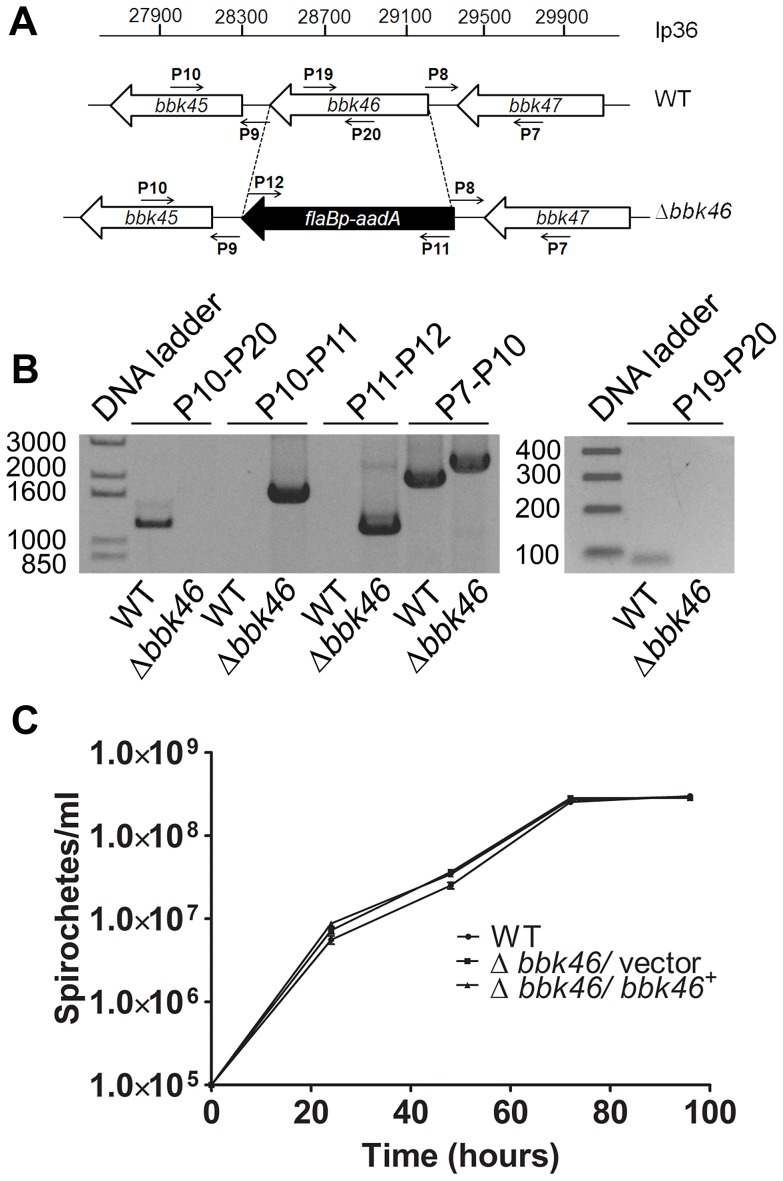
Generation of the Δ*bbk46* mutant and genetic complemented clones in *B. burgdorferi*. (A) Schematic representation of the wild-type (WT) and Δ*bbk46* loci on lp36. The sequence of the entire *bbk46* open reading frame was replaced with a *flaB*_p_-*aadA* antibiotic resistance cassette [37,82]. Locations of primers for analysis of the mutant clones are indicated with small arrows and labels P7-P12, P19 and P20. Primer sequences are listed in Table 5. (B) PCR analysis of the Δ*bbk46* mutant clone. Genomic DNA isolated from WT and Δ*bbk46/* vector spirochetes served as the template DNA for PCR analyses. DNA templates are indicated across the bottom of the gel image. The primer pairs used to amplify specific DNA sequences are indicated at the top of the gel image and correspond to target sequences as shown in A. Migration of the DNA ladder in base pairs is shown to the left of each image. (C) *In vitro* growth analysis of mutant clones. A3-68ΔBBE02 (WT), *bbk46*::*flaB*_p_-*aadA*/ pBSV2G (Δ*bbk46*/ vector) and *bbk46*::*flaB*_p_-*aadA*/ pBSV2G-*bbk46* (Δ*bbk46*/ *bbk46^+^*) spirochetes were inoculated in triplicate at a density of 1×10^5^ spirochetes/ml in 5 ml of BSKII medium. Spirochete densities were determined every 24 hours under dark field microscopy using a Petroff-Hausser chamber over the course of 96 hours. The data are represented as the number of spirochetes per ml over time (hours) and is expressed as the average of 3 biological replicates. Error bars indicate the standard deviation from the mean.

**Figure 6:**
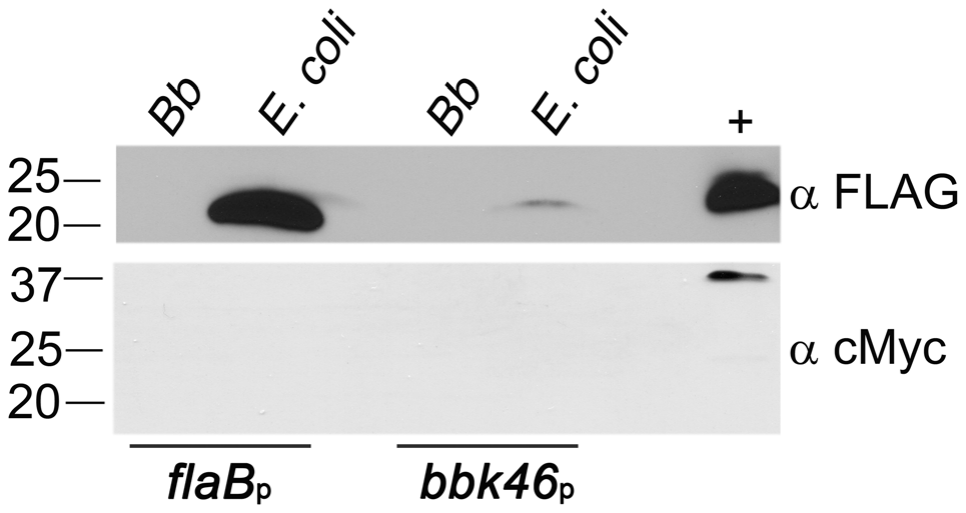
BBK46 protein production is detectable in *E. coli* but not in *B. burgdorferi*. Immunoblot analysis of total protein lysate prepared from 1.5×10^8^ *B. burgdorferi* Δ*bbk46* (*Bb*) or *E. coli* harboring either pBSV2G *flaB*_p_-*bbk46*-*FLAG*-*cMyc* (*flaB*_p_) or pBSV2G *bbk46*_p_-*bbk46*-*FLAG*-*cMyc* (*bbk46*_p_). Protein lysates were separated by SDS-PAGE and immunoblots performed using anti-FLAG monoclonal antibodies (α FLAG) and anti-cMyc monoclonal antibodies (α cMyc). 300 ng of purified PncA-FLAG [23] and GST-BmpA-cMyc [78] proteins served as positive controls (+) for each antibody. The positions of markers to the left of the panel depict protein standard molecular masses in kilodaltons.

As a putative member of the P37 immunogenic lipoprotein family, BBK46 is predicted to localize to the spirochete outer surface and to be immunogenic during mammalian infection. Therefore, recombinant BBK46, lacking the first 32 amino acids that are predicted to comprise the signal sequence for the lipoprotein, was produced in *E. coli* as an N-terminal fusion to glutathione *S*-transferase (GST). To assess the immunogenicity of the BBK46 protein, immunoblot analysis was performed using purified rGST-BBK46 probed with mouse immune serum collected 21 days post inoculation with 1×10^4^ wild-type *B. burgdorferi.* The rGST-BBK46 protein was found to be non-immunoreactive with mouse immune serum, in contrast to the control antigen BmpA (Figure S2). These data suggest that, if produced in *B. burgdorferi*, BBK46 is not an immunoreactive antigen. However, these data do not rule out the possibility that the immunogenic epitope is not present or available in the recombinant protein produced in *E. coli*.

**The *bbk46* gene is dispensable for *B. burgdorferi* infection in mice.** *In vitro* growth analysis demonstrated that the *bbk46* mutant and complemented clones had no detectable *in vitro* phenotypes (Figure 5C). Initial characterization of the role of *bbk46* in mouse infectivity suggested that this gene may contribute to spirochete persistence in mouse tissues. However, this phenotype was subsequently attributed to the loss of lp28-1 from the *bbk46*/vector mutant clone [27–29,47,48], rather than the *bbk46* gene itself. The Δ*bbk46*/vector *B. burgdorferi* clone was re-derived and verified to contain all of the plasmids of the parent clone. Therefore, to examine the role of *bbk46* in mouse infectivity, groups of 9 or 6 C3H/HeN female mice were needle inoculated with 1×10^4^ Δ*bbk46*/vector or Δ*bbk46*/*bbk46* ^+^ spirochetes, respectively. Three weeks post inoculation the mice were assessed for *B. burgdorferi* infection by serology and reisolation of spirochetes from the ear, bladder and joint tissues. All of the mice inoculated with the Δ*bbk46*/vector clone were seropositive for anti-*B. burgdorferi* antibodies and positive for spirochete reisolation from all tissues examined (Table 4). As expected, 5 out 6 mice inoculated with Δ*bbk46*/*bbk46* ^+^ complemented spirochetes demonstrated no defect in mouse infectivity (Table 4). Together these findings suggest that the *bbk46* gene is not required for *B. burgdorferi* needle inoculation of mice at a dose of 1×10^4^ spirochetes.

## Discussion

In this study we have successfully adapted and applied for the first time an IVET-based genetic screen for use in *B. burgdorferi* for the purpose of identifying spirochete genes that are expressed during mammalian infection. Historically, genetic manipulation of low passage, infectious *B. burgdorferi* has been challenged by the low transformation frequencies of these spirochetes, preventing application of classic *in vivo* genetic screening techniques such as *in vivo* expression technology (IVET) and signature-tagged mutagenesis (STM) [49] to identify *B. burgdorferi* genetic elements important for pathogenicity. However, advances in the understanding of the *B. burgdorferi* restriction modification systems that inhibit transformation [34,50–53] have recently allowed construction and characterization of a comprehensive STM mutant library in infectious *B. burgdorferi* [54]. The foundation for our strategy for development of IVET in *B. burgdorferi* was based upon the spirochete's requirement of lp25 for both restriction modification and virulence functions. Spirochetes lacking lp25 are highly transformable but non-infectious in mice [27–29,34]. Restoration of the lp25-encoded *pncA* gene to lp25^−^ spirochetes restores wild-type infectivity [24] but maintains high transformation frequency. At the time of the development of the pBbIVET system the true start codon of the *pncA* gene was not defined; therefore, the promoter-less *pncA* gene construct in the pBbIVET plasmid used an engineered AUG start codon and was missing the first 24 nucleotides of the now defined *pncA* ORF [23]. Furthermore, this construct was purposefully designed without a ribosome binding site (RBS) and was dependent upon the cloned *B. burgdorferi* DNA fragments to contain both a promoter and a functional RBS. Although we acknowledge that this requirement may have limited the number of clones identified in our screen, during development of the BbIVET system we found that inclusion of an RBS sequence in the promoterless *pncA* construct resulted in vector-driven PncA production in the absence of a promoter. Thus, in order to reduce the possibility of recovering false positive clones, the pBbIVET system was designed without an RBS. The enzyme Tsp509I was selected to generate the DNA fragments for the pBbIVET library because the AATT restriction site of this enzyme is present approximately every 58 bp in the *B. burgdorferi* B31 genome. However, it is possible that DNA fragments generated with this enzyme will not result in sequences that contain a 3′ RBS appropriately distanced from the start codon of the *pncA* ORF, thereby limiting the number of clones identified in the screen.

Screening of a 15,000 clone *B. burgdorferi* genomic library in mice identified 289 DNA sequences from across all 22 *B. burgdorferi* replicons capable of promoting *pncA* expression resulting in an infectious phenotype. It is likely that the BbIVET screen did not achieve saturation because the number of clones analyzed was only estimated to cover the *B. burgdorferi* genome one time, under the assumption that each cloned DNA fragment in the library was unique. Analysis of the pBbIVET library in *B. burgdorferi* suggested that the library was composed of 15,000 unique clones. However, because only a small fraction of the library was examined for the sequences of the DNA fragment inserts, our findings do not rule out the potential that the library was composed of less than 15,000 non-identical clones and therefore, may represent less than 1X coverage of the genome. Of the 175 mice infected with the pBbIVET library, 10% resulted in reisolation of a single clone, 62% resulted in reisolation of two to five unique clones, and 28% resulted in reisolation of six to eleven unique clones. Furthermore, 57% of the 289 *Bbive* sequences were only recovered once; whereas, 39% of the sequences were recovered two to five times and 4% of the sequences were recovered six to twelve times. These data are indicative of the amount of redundancy in the screen and suggest that although the screen may not have been representative of the entire *B. burgdorferi* genome, a large percentage of mice became infected with multiple clones and many of the *Bbive* sequences were recovered more than once.

We found that 71 of the *Bbive* sequences mapped to canonical promoter positions upstream of annotated open reading frames in the *B. burgdorferi* genome. Unexpectedly, the well characterized *in vivo*-expressed *ospC* promoter was not among these sequences. However, the *ospC*_p_ was successfully recovered in our functional validation of the BbIVET system, suggesting that the BbIVET screen had not reached complete saturation of the genome and with further screening of the BbIVET library the *ospC*_p_ sequence may be recovered. Alternatively, given that *ospC* expression is known to be down-regulated after the initial stages of infection [11,55–58] it is possible that in the context of a mixed infection individual pBbIVET clones carrying the *ospC*_p_ lack a fitness advantage due to decreased expression three weeks post inoculation and may not be recovered in our screen. This explanation may appear to conflict with the findings reported herein that *ospC* expression is high at three weeks post inoculation and the *ospC*_p_ served as a robust positive control promoter for the BbIVET system. However, down-regulation of *ospC* expression at this time point in infection is a stochastic process that occurs at the level of the individual spirochete and does not occur simultaneously across the entire population [57]. Although at the population level the *ospC*_p_ is expressed at this time point in our studies, in the context of the BbIVET screen individual clones carrying the *ospC*_p_ may express reduced amount of *pncA* and may be out competed by other BbIVET clones carrying stronger promoters.

A subset of the genes identified in the BbIVET screen included known *in vivo*-expressed genes, which provided validation that our genetic system was working as expected and was sufficiently powerful. The screen recovered the promoter for genes *bba36* (*Bbive175*), *bba57* (*Bbive271*), *bbb27* (*Bbive240*), *bbj34* (*Bbive267*), *bbj36* (*Bbive171*), *bbj51* (*Bbive269*), *bb0213* (*Bbive15*) and *bb0760* (*Bbive94*), all of which have been shown previously to be expressed during mammalian infection [11]. Furthermore, *bba57* was recently reported to be up-regulated *in vivo* and to contribute to pathogenesis in the mouse [59]. The *bptA* gene encodes a function that has been shown to be required for *B. burgdorferi* survival in the tick and to contribute to mouse infectivity [30,31]. In addition, *Bbive14*, *58*, *232*, *84*, *269*, *295* and *77* are associated with genes that have been shown to be up-regulated in *in vivo*-like conditions and/or gene products that are immunogenic in humans and mice [5,8,60,61]. Notably, few *in vivo*-expressed candidate genes identified using BbIVET were previously observed to be up-regulated in mammalian host-adapted spirochetes derived from growth within rat dialysis membrane chambers (DMCs). Genes identified in our analyses that have also been detected by microarray analysis of DMC grown spirochetes include *bba36* [8,10], *bbj51* [7,8], *bb0551*, *bbm28* [8], *bb0495,* and *bb0660* [7]. The results of the DMC microarray studies are reported as genes that are significantly up-regulated in DMC-derived spirochetes relative to spirochetes grown *in vitro*; whereas, the BbIVET screen does not distinguish between genes that are specifically induced *in vivo* and genes that are expressed both *in vitro* and *in vivo*. Furthermore, the environmental cues within the DMCs may not fully reflect those experienced by *B. burgdorferi* during an active infection. Finally, the BbIVET system specifically selects for promoters that are capable of driving expression of *pncA* allowing the spirochetes to survive throughout a three week mouse infection. Together, these technical and biological differences between the DMC microarray and BbIVET screen likely contributed to the distinct results obtained from the two methods of gene expression analysis. In addition, few genes that have been previously established to be RpoS-regulated *in vitro* and/or within DMCs [10,62] were identified by the BbIVET screen. RpoS-regulated genes *bba36*, *bba57*, *bb0265* and *bbh01* [10,62] were among the *in vivo*-expressed *Bbive* candidate genes. Similarly, only one putative BosR-regulated gene, *bb0592* [63], was identified in the BbIVET screen. Although it is unclear why only a small number of known RpoS-regulated promoters were recovered, the recently identified AT-rich BosR binding site [63] contains the restriction site for the Tsp509I restriction enzyme used to generate the BbIVET library. Therefore, it is possible that the BosR binding sites were subject to cleavage by Tsp509I, perhaps resulting in a limited number of DNA fragments that contained BosR-dependent promoters.

The BbIVET screen was carried out in such a way that both DNA fragments that are expressed *in vitro* and *in vivo*, as well as those fragments that are specifically induced *in vivo*, could be recovered. Therefore, it was not surprising that genes encoding cell division, DNA replication, energy metabolism, protein synthesis and transcription functions were identified, all of which are likely functions essential for spirochete growth under all condition. These findings were consistent with those categories of genes not recovered by genome-wide transposon mutagenesis, suggesting that these genes encode essential functions [54]. The BbIVET screen identified genes that encode proteins in functional categories that may contribute to *B. burgdorferi* infectivity and pathogenesis including, putative lipoproteins, motility and chemotaxis proteins, transport proteins and proteins of unknown function. Similarly, transposon mutagenesis analysis indicated that motility and chemotaxis genes as well as transport genes are important for *B. burgdorferi* survival in the mouse [54].

Linear plasmid 36 is known to be critical for *B. burgdorferi* survival in the mouse; however, the genes on lp36 that contribute to this requirement have not been fully characterized [37]. The recently published comprehensive STM study suggests that many of the genes encoded on lp36 participate in *B. burgdorferi* infectivity [37,54]. BbIVET identified gene *bbk46* on lp36. We found that *bbk46* was expressed both *in vitro* and *in vivo.* However, *bbk46* expression was dramatically induced in spirochetes isolated from infected mouse tissues as compared to spirochetes grown *in vitro*, suggesting a possible role for this gene in *B. burgdorferi* infectivity. Conversely, no role for *bbk46* in *B. burgdorferi* mouse infection was detected. Consistent with lack of identification of *bbk46* as an RpoS-regulated genes in previous studies of the RpoS regulon [10,64], control of *bbk46* expression was found to be RpoS-independent under *in vitro* growth conditions that typically induce expression of *rpoS* regulated genes [4,8,42,62]. These findings highlight the power and uniqueness of the IVET-based approach for identification of *B. burgdorferi in vivo*-expressed genes, which might not be discovered using other genome-wide gene expression methods. Surprisingly, BBK46 protein was not detected in spirochetes expressing FLAG epitope tagged *bbk46* under the control of the putative native promoter or the constitutive *flaB* promoter. Moreover, sera from *B. burgdorferi* infected mice were non-immunoreactive against recombinant BBK46 protein. In support of these data, no peptide corresponding to BBK46 has been detected in genome-wide proteome analysis of *B. burgdorferi* under different environmental conditions [65]. Our findings suggest that despite high gene expression, the encoded BBK46 protein is produced at low levels in the spirochete and/or BBK46 is rapidly turned over in the cell. Alternatively, *bbk46* may function as an RNA. The molecular nature of the functional product of *bbk46* is currently under investigation.

In conclusion, we have developed and applied the IVET technology to *B. burgdorferi* to identify spirochete genes expressed during mammalian infection. This represents the first use of this system in *B. burgdorferi*. The power of BbIVET was validated by identification of a subset of genes that have been demonstrated previously to be upregulated *in vivo* as well as unique *B. burgdorferi in vivo*-expressed genes that had not been recognized previously. Further analysis of the *B. burgdorferi in vivo*-expressed genes identified herein will contribute to advancing understanding of the molecular mechanisms of *B. burgdorferi* survival and pathogenicity in the mammalian host.

## Materials and Methods

**Ethics statement.** The University of Central Florida is accredited by the International Association for Assessment and Accreditation of Laboratory Animal Care. Protocols for all animal experiments were prepared according to the guidelines of the National Institutes of Health and were reviewed and approved by the University of Central Florida Institutional Animal Care and Use Committee (Protocol numbers 09-38 and 12–42).

**Bacteria clones and growth conditions.** All *B. burgdorferi* clones used were derived from clone B31 A3. Clone A3 68-1, which lacks lp25 and lp56 [35] was used for the pBbIVET library. The B31 A3 wild-type and *rpoS*::*kan B. burgdorferi* clones [71] were used for gene expression experiments. All low-passage *B. burgdorferi* mutant and complemented clones generated herein were derived from infectious clone A3-68ΔBBE02, which lacks cp9, lp56 and gene *bbe02* on lp25 [53]. *B. burgdorferi* was grown in liquid Barbour-Stoenner-Kelly (BSK) II medium supplemented with gelatin and 6% rabbit serum [72] and plated in solid BSK medium as previously described [73,74]. All spirochete cultures were grown at 35°C supplemented with 2.5% CO2. Kanamycin was used at 200 µg/ml, streptomycin was used at 50 µg/ml and gentamicin was used at 40 µg/ml, when appropriate. All cloning steps were carried out using DH5α *E. coli*, which were grown in LB broth or on LB agar plates containing 50 µg/ml kanamycin, 300 µg/ml spectinomycin or 10 µg/ml gentamicin.

**Generation of the pBbIVET plasmid.** The promoterless *pncA* gene was amplified from *B. burgdorferi* B31 genomic DNA using primers 1 and 2 (Table 5) and Taq DNA polymerase (New England Biolabs). The EcoRI/XbaI-digested *pncA* fragment was cloned into EcoRI/XbaI-linearized plasmid pBSV2*TT [23], creating plasmid pBbIVET. The *in vivo*-expressed *ospC* promoter with EcoRI ends was amplified from *B. burgdorferi* B31 genomic DNA using primers 3 and 4 (Table 5) and cloned into the EcoRI-cut, Antarctic phosphatase-treated (New England Biolabs) pBbIVET plasmid in front of the promoterless *pncA* gene, resulting in plasmid pBbIVET *ospC*_p_. All plasmids were analyzed and verified by restriction digest and sequence analysis. The pBbIVET and pBbIVET *ospC*_p_ plasmids were each transformed by electroporation into A3 68-1 [35] as described [37] and transformants selected in solid BSK medium containing kanamycin and confirmed by PCR using primers 1 and 2 (Table 5). Total genomic DNA was prepared from PCR-positive clones and screened for the presence of the *B. burgdorferi* plasmid content [71]. The clones that retained the plasmid content of the parent clone were used in further experiments.

**Table 5:** List of primers used in this study.

Primer number	Designation	Sequence (5′ – 3′)^a^
1	pncA 5′ EcoRI A	cggaattcatgGCACTTATTTTAATAGATATAC
2	pncA 3′ XbaI	gctctagaTTATATATTAAGCTTACTTTGGCTG
3	ospC prom 5′ EcoRI	cggaattcTTCTTTTTCATTAATTTGTGCCTCC
4	ospC prom 3′ EcoRI	cggaattcTTAATTTTAGCATATTTGGCTTTGCTTATGTCG
5	pUC18R BSV2	AGCGGATAACAATTTCACACAG
6	pncA prom 3′ seq	ACTGTTAGATACTGGCAAAGTGCC
7	bbk46Fup500	GTTCTTTTATGGAGCAAGCAACTAA
8	bbk46Rup500	CGGAAGCCACAAGAGGCGACAGACACTATCTTAGTACCTCTTCTTAGAATCTG
9	bbk46Fdown500	GGCGAGATCACCAAGGTAGTCGGCAAATAAATAATACTAATCTTAGATAGCTCAGCTTT
10	bbk46Rdown500	CTAGCTTCACTAGTTTCCCTAGA
11	flaBpaadA F	TGTCTGTCGCCTCTTGTG
12	flaBpaadA R	TTATTTGCCGACTACCTTGGTG
13	K465′kpn1fwd	cggggtaccCTTCCAGTGTAGGCTTTAGTTT
14	K463′FLAGrev	TTAtttatcatcatcatctttataatcTGCCTCAACTGCCTTTCTC
15	K465′FLAGfwd	gattataaagatgatgatgataaaTAAAATGCTTCAAAGGAAAATTATGAATGG
16	K463′C-mycSalIrev	acgcgtcgacTTAcagatcttcttcagaaataagtttttgttcATAAGCAGCTTCATATGCTTTATTT
17	K465′PCR3fwd	CGGGGTACCCTTCCAGTGTAG
18	K463′PCR3rev	ACGCGTCGACTTACAGATCTTCTTCAGAAATA
19	Lp3629018F	AGCATTATTTGTACTTCTAGGC
20	Lp3629013R	ACATACTAGACAACAACAAGTC
21	flaBF3	GCATTAACGCTGCTAATCTTAG
22	flaBR3	GCATTAATCTTACCAGAAACTCC
23	recA F	AATAAGGATGAGGATTGGTG
24	recA R	GAACCTCAAGTCTAAGAGATG
25	ospC1 F	ACGGATTCTAATGCGGTTTTACCT
26	ospC1 R	CAATAGCTTTAGCAGCAATTTCATCT
27	flaBp 5′ KpnI	gggggtaccTGTCTGTCGCCTCTTGTGGCT
28	flaBp 3′ BamHI	gggggatccGATTGATAATCATATATCATTCCT
29	bbk46+S 5′ BamHIF	cgggatccATGAATTTAATTGCTAAATTATTTATTTTATCCAC
30	bbk46-S 5′ BamHIF	cgggatcc ATGTGTAACCTATATGATAATCTTGCAGAC
31	bbk46 3′ XhoIR	ccgctcgag TTAATAAGCAGCTTCATATGCTTTATTTAG

a Lowercase indicates all non-*B. burgdorferi* sequence.

**Generation of the BbIVET library.** Total genomic DNA was isolated from a 250 ml culture of *B. burgdorferi* B31 clone A3 grown to a density 1×10^8^ spirochetes/ml using the Qiagen genomic DNA buffer set and Genomic-tip 500/G, according to the manufacturer's protocol (Qiagen). A3 genomic DNA was partially digested with Tsp509I (New England Biolabs). The partial digests were electrophoretically separated on a 0.8% agarose gel and the 300 to 500 bp range of DNA fragments extracted and ligated in a 1∶1 molar ratio with EcoRI-digested and Antarctic phosphatase-treated pBbIVET. Library ligations were electroporated into *E. coli* Top10 cells (Life Technologies) and transformants selected on LB agar containing 50 µg/ml kanamycin, resulting in approximately 30,000 independent clones. Plasmid DNA was isolated from these cells and 20 µg aliquots of the plasmid library were transformed by electroporation into *B. burgdorferi* A3 68-1, as previously described [74]. One fifth of each transformation was plated on solid BSK medium containing kanamycin. *B. burgdorferi* pBbIVET colonies were verified to contain *B. burgdorferi* DNA fragments by PCR using primers 5 and 6 (Table 5) and the number of transformants recovered quantitated. The approximately 15,000 *B. burgdorferi* clones recovered over 40 transformations were stored in aliquots of pools of approximately 100 BbIVET clones each in 25% glycerol at −80°C.

**Selection of *B. burgdorferi* clones having *in vivo*-expressed DNA fragments.** Each BbIVET pool (∼100 clones) was grown in 10 ml of fresh BSKII medium to a density of 1×10^8^ spirochetes/ml. In groups of approximately 20 animals, 144 6–8 week old C3H/HeN female mice were each inoculated (80% intraperitoneal and 20% subcutaneous) with a dose 1×10^6^ spirochetes of a unique pool of ∼100 BbIVET clones, under the assumption that each clone was present at dose 1×10^4^ spirochetes. A fraction of each inoculum was plated on solid BSK medium and colonies screened for the presence of virulence plasmid lp28-1. Three weeks post inoculation, spirochetes were reisolated from ear, heart, bladder and joint tissues in 10 ml BSKII medium containing 20 µg/ml phosphomycin (Sigma), 50 µg/ml rifampicin (Sigma) and 2.5 mg/ml amphotericin B (Sigma) in 0.2% dimethyl sulfoxide (Sigma). Total genomic DNA was isolated from each spirochete cultures using the Wizard genomic DNA purification kit (Promega) and transformed into chemically competent *E. coli* DH5α cells and colonies selected on LB agar containing kanamycin to recover the pBbIVET plasmids. Twenty four transformants were chosen at random from each plasmid rescue and colony PCR performed using primers 5 and 6 (Table 5) to amplify the *in vivo*-expressed DNA fragment. PCR products were subsequently digested with a cocktail of restriction enzymes (DraI, SspI and AseI) and visualized on a 1% agarose gel. Approximately 14,000 *E. coli* clones were analyzed in this manner. All unique BbIVET fragments, as determined by the restriction digest pattern (Figure S1), were analyzed by direct sequencing of the PCR product using primer 5. Each individual sequence was identified by blastn analysis and mapped to its location in the *B. burgdorferi* B31 genome.

**Deletion of *bbk46*.** We used a PCR-based overlap extension strategy to delete the *bbk46* gene. A spectinomycin/ streptomycin resistance cassette, *flaBp-aadA* [75] with blunt ends, was amplified from genomic DNA isolated from clone Δ*guaAB* [35] using Phusion High-fidelity DNA polymerase (Thermo Scientific) and primers 11 and 12 (Table 5). The 500 bp flanking region upstream of the *bbk46* ORF was amplified from the *B. burgdorferi* B31 clone A3 genomic DNA using the Phusion High-fidelity DNA polymerase and primers 7 and 8 (Table 5). This introduced a 25 bp sequence at the 3′ end of this fragment that was complementary to the 5′ end of the *flaBp-aadA* cassette. Similarly, the 500 bp flanking region downstream of the *bbk46* ORF was amplified using the primers 9 and 10 (Table 5), which introduced a 5′ sequence of 30 bp that was complementary to the 3′ end of the resistance cassette. The PCR products from the above 3 reactions were mixed in equal volumes and used as a template for a fourth amplification reaction using Phusion High-fidelity DNA polymerase and primers 7 and 10 (Table 5) in order to generate a product containing the resistance cassette flanked by the 500 bp sequences upstream and downstream of the *bbk46* ORF. This product was ligated with linear pCR-Blunt using a Zero Blunt PCR cloning Kit (Life technologies), yielding the allelic exchange plasmid pCR-Blunt*-*Δ*bbk46-flaBp-aadA*. *B. burgdorferi* A3-68ΔBBE02 was transformed with 20 µg of pCR-Blunt*-*Δ*bbk46-flaBp-aadA* purified from *E. coli* as previously described [37]. Streptomycin-resistant colonies were confirmed to be true transformants by PCR using primer pairs 7 and 10 and 11 and 12 (Table 5). Positive Δ*bbk46-flaBp-aadA* clones were screened with a panel of primers [71] for the presence of all of the *B. burgdorferi* plasmids of the parent A3-68ΔBBE02 clone [53], and a single clone was selected for further experiments.

**Complementation of the** Δ***bbk46*** **mutant.** A PCR-based overlap extension strategy was used to create a DNA fragment encompassing the *bbk46* gene and putative upstream promoter sequence with the introduction of a FLAG epitope tag immediately upstream of the putative premature stop codon and a cMyc epitope tag immediately upstream of the downstream stop codon. This was done by using Phusion High-fidelity DNA polymerase (New England Biolabs) and the primers pairs 13 and 14, 15 and 16, and 17 and 18 (Table 5). A KpnI restriction site was introduced at the 5′ end of this fragment and a SalI site at the 3′ end. The KpnI+SalI-digested PCR product was ligated into KpnI+ SalI-digested *B. burgdorferi* shuttle vector pBSV2G [76] and cloned in *E. coli*. The pBSV2G *bbk46*_p_-*bbk46-FLAG-cMyc* plasmid structure and sequence were confirmed by restriction digest and DNA sequence analysis. In addition, a 400 bp DNA fragment encompassing the *flaB* promoter with KpnI and BamHI ends was amplified from B31 A3 genomic DNA using primers 27 and 28 (Table 5). The KpnI+BamHI-digested PCR product was ligated into KpnI+ BamHI-digested *B. burgdorferi* shuttle vector pBSV2G [76]. The *bbk46*-*FLAG*-*cMyc* gene without the putative *bk46* promoter sequence and with BamHI and SalI ends was amplified from pBSV2G *bbk46*_p_-*bbk46-FLAG-cMyc* plasmid DNA using Phusion High-fidelity DNA polymerase (New England Biolabs) and primers 29 and 18 (Table 5). The BamHI+SalI-digested PCR product was ligated into BamHI+SalI-digested pBSV2G*flaB*_p_ and cloned in *E. coli*. The pBSV2G *flaB*_p_-*bbk46-FLAG-cMyc* plasmid structure and sequence were confirmed by restriction digest and DNA sequence analysis. The Δ*bbk46* mutant was transformed with 20 µg of pBSV2G *bbk46*_p_-*bbk46-FLAG-cMyc,* pBSV2G *flaB*_p_-*bbk46-FLAG-cMyc* or pBSV2G alone isolated from *E. coli* and positive transformants selected as previously described [37,77]. The clones that retained the *B. burgdorferi* plasmid content of the parent clone were selected for use in further experiments.

**Immunoblot analysis of BBK46-FLAG-cMyc.** Production of the BBK46-FLAG-cMyc protein was examined in both *E. coli* and *B. burgdorferi* carrying pBSV2G *bbk46*_p_-*bbk46-FLAG-cMyc* or pBSV2G *flaB*_p_-*bbk46-FLAG-cMyc*. Total *E. coli* protein lysates were prepared from 2×10^9^ cells harvested following overnight growth in LB medium at 37°C with aeration. *E. coli* cells were resuspended and lysed in 200 µl B-PER protein extraction reagent (Pierce), followed by the addition of 200 µl 2× Laemmli sample buffer plus 2-mercaptoethanol (Bio-rad). Total *B. burgdorferi* protein lysates were prepared from 2×10^9^ spirochetes harvested at mid-log phase. The spirochetes were washed twice in 1 ml cold HN buffer (50 mM Hepes, 50 mM NaCl, pH 7.4) and lysed in 200 µl B-PER protein extraction reagent (Thermo Scientific), followed by the addition of 200 µl 2× Laemmli sample buffer plus 2-mercaptoethanol (Bio-rad). 30 ml of each protein lysate (∼1.5×10^8^ cells) were separated by SDS-PAGE and transferred to a nitrocellulose membrane. 300 ng of PncA-FLAG [23] and GST-BmpA-cMyc [78] proteins served as positive controls. Immunoblot analysis was performed using anti-FLAG monoclonal primary antibody (Genscript) diluted 1∶500 in Tris-buffered saline, pH 7.4 and 0.5% Tween20 (TBST) and goat anti-mouse IgG+IgM-HRP secondary antibody (EMD Millipore) diluted 1∶10,000 in TBST and the signal detected using SuperSignal West Pico chemluminescent substrate kit (Thermo Scientific). The membrane was then stripped using 0.2M NaOH, reblocked using 5% skim milk in TBST and probed with anti-cMyc primary antibody (Genscript) diluted 1∶500 in TBST and goat anti-mouse IgG+IgM-HRP (EMD Millipore) and visualized as described above.

**Cloning, purification and seroreactivity analysis of rGST-BBK46.** An in-frame glutathinone *S*-transferase (GST)-BBK46 fusion protein lacking the putative BBK46 signal sequence was generated using primers 30 and 31 (Table 5) and purified, as previously described [78]. Approximately 1 µg of GST-BBK46 was separated by SDS-PAGE, transferred to a nitrocellulose membrane and analyzed by immunoblot for seroreactivity using immune serum collected 3 weeks post inoculation from mice infected with wild-type *B. burgdorferi* as previously described [78]. Controls included 1 µg of GST alone and total protein lysates generated from BL21 *E. coli*, *B. burgdorferi* B31 A3 and *E. coli* expressing *B. burgdorferi bmpA* [37] prepared as described above. The membrane was stripped as described above and reprobed with anti-GST primary monoclonal antibody (EMD Millipore) diluted 1∶1000 in TBST and goat anti-mouse IgG+IgM-HRP (EMD Millipore) and visualized as described above.

***In vitro* growth analysis.** Wild-type (A3-68ΔBBE02), Δ*bbk46*/vector and Δ*bbk46/bbk46* ^+^ spirochetes were inoculated in triplicate at a density of 1×10^5^ spirochetes/ml in 5 ml of BSK II medium. Spirochete densities were determined every 24 hours under dark field microscopy using a Petroff-Hausser chamber over the course of 96 hours.

**RNA isolation from *in vitro* grown spirochetes.** To obtain *in vitro* grown log phase spirochetes, wild-type (B31 A3) spirochetes were grown in triplicate in 5 ml of BSKII medium pH 7.5 at 35°C to a density of 3×10^7^ spirochetes/ml. To obtain stationary phase, temperature-shifted spirochetes, wild-type (B31 A3) spirochetes were grown in triplicate in 5 ml of BSKII medium pH 7.5 at 35°C to a density of 3×10^7^ spirochetes/ml, transferred to 25°C for 48 hours and then returned to 35°C for an additional 24–36 hours to a density of 2×10^8^ spirochetes/ml. A total of 1×10^7^ spirochetes were harvested from each culture and total RNA was isolated using TRIzol reagent (Life Technologies) according to the manufacturer's instructions. RNA was resuspended in 100 µl DEPC-treated dH_2_O. RNA was treated with TURBO DNA-free (Life Technologies) to remove any contaminating genomic DNA. 1 µl of Riboguard (40U/µl) RNAse inhibitor (Epicentre) was added to all samples and RNA stored at −80°C.

**RNA isolation from infected mouse tissue.** *B. burgdorferi-*infected mouse bladders (see mouse infection experiments below) were manually macerated on ice using sterile scalpels and transferred to a 2 ml sterile tube containing lysing Matrix D (MP Biomedicals). 1ml of RNA pro solution (FastRNA Pro Green kit, MP Biomedicals) was added to each sample on ice. Tissues were homogenized using a PowerGen High-Throughput Homogenizer (Fisher Scientific) following six cycles of beating for 45 sec and 2 minute incubations on ice. Samples were centrifuged at 13,000 rpm for 5 minutes at 4°C. The upper aqueous phase was transferred to new tubes and incubated for 5 minutes at room temperature. 500 µl of 1-bromo-3-chloropropane (Sigma Aldrich) and 45 µl of 5M sodium acetate were added to each sample and samples were incubated for an additional 5 minutes at room temperature. Samples were centrifuged at 13,000 rpm for 5 minutes at 4°C. The upper aqueous phase was transferred to new tubes and RNA precipitated with the addition of 500 µl of absolute ethanol and 1 µl GlycoBlue (Life technologies). RNA was pelleted by centrifugation at 13,000 rpm for 10 minutes at 4°C. RNA was washed with 70% ethanol in DEPC-treated dH_2_0 and resuspended in 100 µl DEPC-treated dH_2_0. RNA was treated with TURBO DNA-free (Life Technologies) to remove any contaminating genomic DNA. 1 µl Riboguard (40U/µl) RNAse inhibitor (Epicentre) was added to all samples and RNA stored at −80°C.

**Gene expression analysis.** cDNA was synthesized from 1.0 µg of each RNA sample using the iScript cDNA synthesis kit (Bio-Rad) with random primers according to the manufacturer's instructions. Parallel cDNA reactions were carried out in the absence of reverse transcriptase. Real-time quantitative PCR (qPCR) reactions were prepared using 1 µg of each cDNA and iQ SYBR Green Supermix (Bio-Rad). Using an Applied Biosystems 7500 instrument, samples were assayed for the *flaB*, *recA*, *ospC* and *bbk46* transcripts using primers pairs 21 and 22, 23 and 24, 25 and 26, and 19 and 20, respectively (Table 5). Standard curves were generated for each gene target using 100 ng, 10 ng, 1.0 ng, 0.1 ng, and 0.01 ng of B31 A3 *B. burgdorferi* genomic DNA and the amount of each gene transcript calculated. The *recA* transcript was used as the endogenous reference to which the transcripts of the other genes were normalized. The *bbk46* primers were confirmed to be specific for their gene target. Three biological replicate samples were analyzed in triplicate and normalized to *recA* mRNA. The data were reported as the average gene transcript/*recA* transcript for each sample. The amplification of samples lacking reverse transcriptase was similar to that of the no-template control.

**Mouse infection experiments.** Unless otherwise noted, groups of 6–8 week old C3H/HeN female mice (Harlan) were used for all experiments.

*ID_50_ analysis of mammalian-adapted spirochetes.* A single C3H/HeN SCID mouse (Harlan) was inoculated with 2×10^6^ *B. burgdorferi* B31 A3. Two weeks post infection the infected blood was harvested and used to inoculate groups of six wild-type C3H/HeN (Harlan) mice with 100 µl of undiluted infected blood or 100 µl of infected blood diluted 1∶10 or 1∶100 in BSK-H medium. The number of live spirochetes in the infected blood and therefore the actual spirochete dose in the inoculum was determined by plating the blood in solid BSK medium and quantitating the number of colony forming units (Table 1). In addition, groups of six wild-type C3H/HeN mice (Harlan) were inoculated with 5×10^4^, 5×10^3^, 5×10^2^, 5×10^1^ or 5×10^0^, *in vitro* grown spirochetes at mid-log phase. The *in vitro* grown spirochetes were confirmed to harbor all plasmids required for infectivity [71].

*Functional validation of the BbIVET system.* Groups of 6 mice were needle inoculated as described [77] with 1×10^4^ spirochetes of clone A3 68-1 carrying pBbIVET or pBbIVET-*ospC*_p_. Mouse infection was assessed 3 weeks post inoculation by reisolation of spirochetes from ear, bladder and joint tissues as previously described [71,79].

*Gene expression studies.* Three mice were needle-inoculated intradermally under the skin of the upper back with *B. burgdorferi* clone B31 A3 at a dose of 1×10^5^ spirochetes. Three weeks post inoculation mouse infection was determined by serology [71,79] and bladders harvested for RNA isolation.

bbk46 *mutant infectivity studies*. Groups of mice were needle inoculated as described [77] with 1×10^4^ spirochetes with *B. burgdorferi* clones Δ*bbk46*/vector (nine mice) or Δ*bbk46/bbk46* ^+^ (six mice) at a dose of 1×10^4^ spirochetes. The number of spirochetes inoculated into mice was determined using a Petroff-Hausser counting chamber and verified by colony-forming unit (cfu) counts in solid BSK medium. Twelve colonies per inoculum were verified by PCR for the presence of the virulence plasmids lp25, lp28-1 and lp36 in at least 90% of the individuals in the population. Further, total plasmid content of each inoculum was confirmed to be as expected [37,71,77]. Mouse infection was assessed three weeks post inoculation by serology using total *B. burgdorferi* lysate, as previously described [37]. Spirochetes were reisolated from the ear, bladder and joint tissues, as previously described [37].

## Supporting Information

Figure S1 **Representative restriction digest analysis of individual pBbIVET plasmids rescued in *E. coli*.** Colony PCR to amplify the *in vivo-*expressed DNA fragment was performed on a random subset of twenty four *E. coli* transformants carrying the rescued pBbIVET plasmids from infected mouse tissues. The PCR products were digested with a cocktail of the restriction enzymes DraI, SspI and AseI and separated on a 1% agarose gel. Numbers across the top of each image identify each non-identical restriction digestion pattern detected for the amplified pBbIVET DNA fragments. Representative data from two mouse tissues (A) and (B) are shown. Migration of the DNA ladders is shown in base pairs on both sides of each image. NTC, PCR no template control.
(TIF)

Figure S2 **The BBK46 protein is non-immunogenic in mice.** Recombinant GST-BBK46 and GST alone produced in and purified from *E. coli*, along with total protein lysate from *E. coli* and *B. burgdorferi* (*Bb* lysate) and *E. coli* producing the *B. burgdorferi* antigen BmpA (+) were separated by SDS-PAGE and transferred to a nitrocellulose membrane. Immunoblot analysis was performed using immune serum collected from mice infected with wild-type *B. burgdorferi* and anti-GST monoclonal antibodies (α GST). The positions of markers to the left of the panel depict protein standard molecular masses in kilodaltons.
(TIF)
