# Supplementary figures and images for: In Vivo Expression Technology Identifies a Novel Virulence Factor Critical for Borrelia burgdorferi Persistence in Mice
Source: PLoS Pathog. 2013 Aug 29;9(8):e1003567. doi: 10.1371/journal.ppat.1003567 (PMC3757035; doi:10.1371/journal.ppat.1003567)

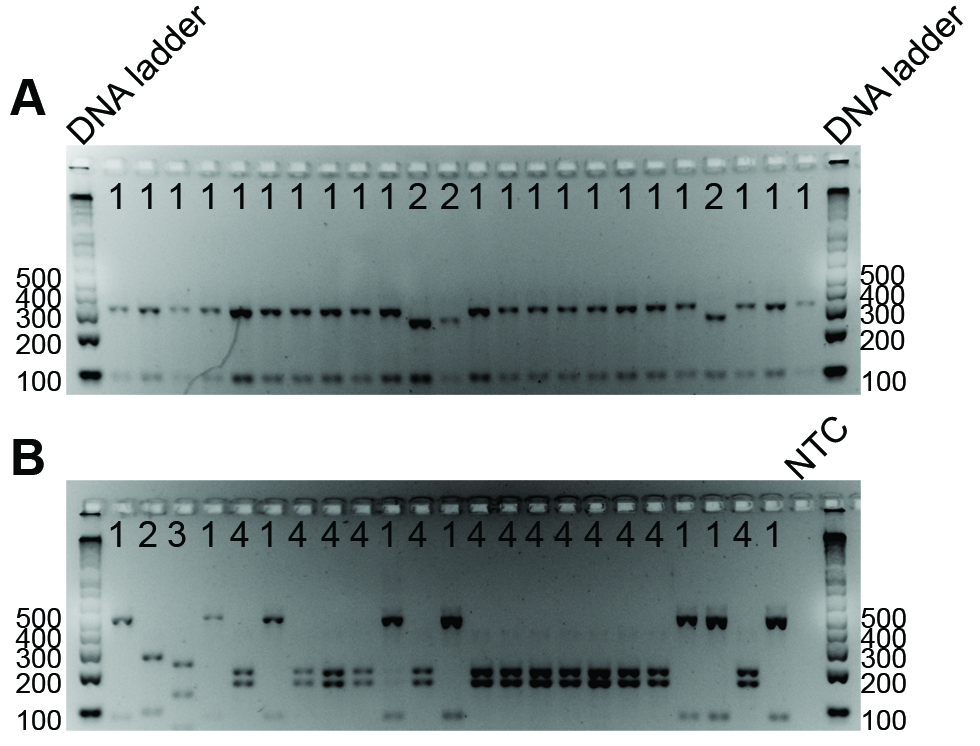

Supplement: Figure S1 — Representative restriction digest analysis of individual pBbIVET plasmids rescued in E. coli . Colony PCR to amplify the in vivo-expressed DNA fragment was performed on a random subset of twenty four E. coli transformants carrying the rescued pBbIVET plasmids from infected mouse tissues. The PCR products were digested with a cocktail of the restriction enzymes DraI, SspI and AseI and separated on a 1% agarose gel. Numbers across the top of each image identify each non-identical restriction digestion pattern detected for the amplified pBbIVET DNA fragments. Representative data from two mouse tissues (A) and (B) are shown. Migration of the DNA ladders is shown in base pairs on both sides of each image. NTC, PCR no template control. (TIF) [file ppat.1003567.s001.tif]

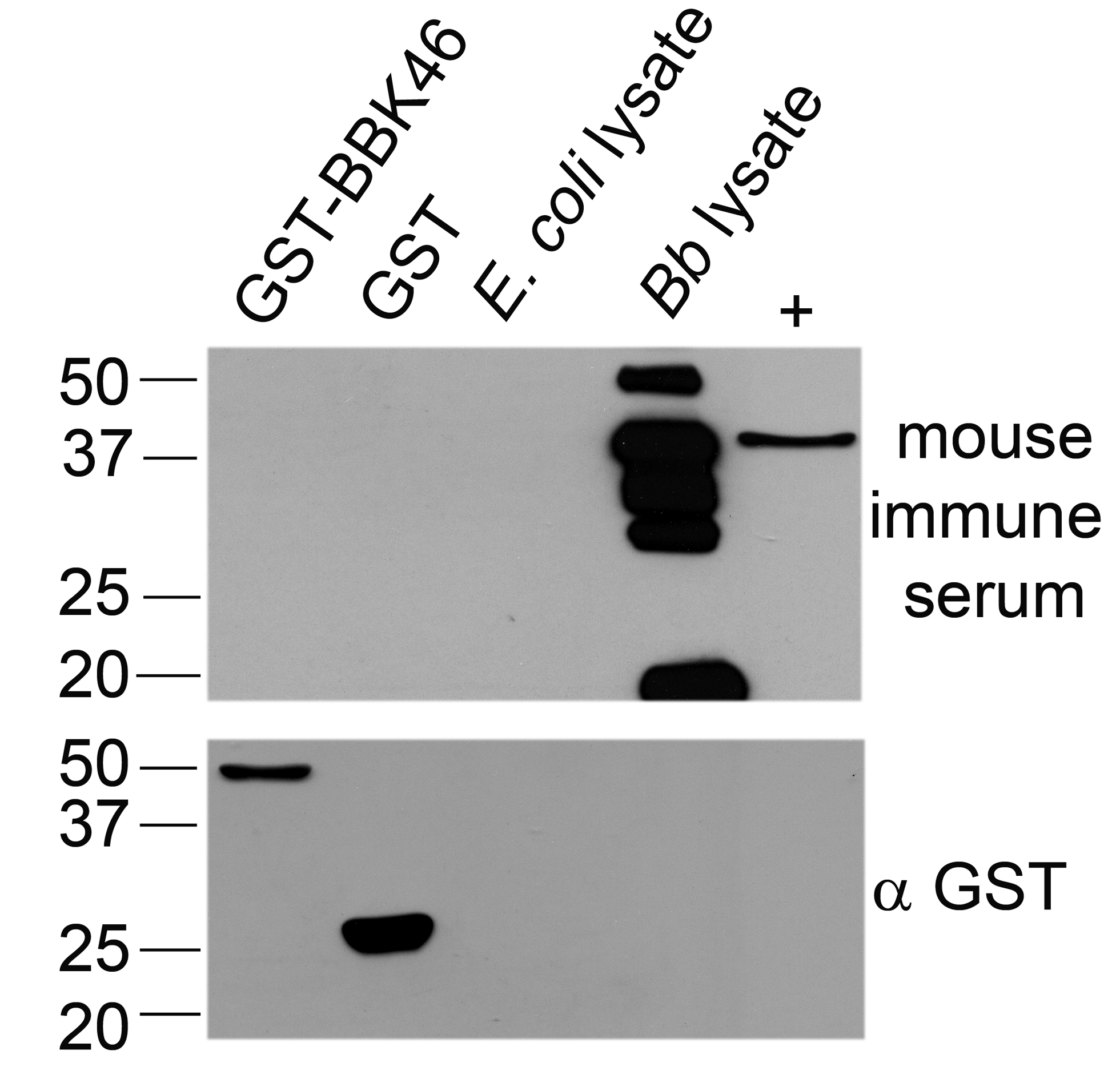

Supplement: Figure S2 — The BBK46 protein is non-immunogenic in mice. Recombinant GST-BBK46 and GST alone produced in and purified from E. coli, along with total protein lysate from E. coli and B. burgdorferi (Bb lysate) and E. coli producing the B. burgdorferi antigen BmpA (+) were separated by SDS-PAGE and transferred to a nitrocellulose membrane. Immunoblot analysis was performed using immune serum collected from mice infected with wild-type B. burgdorferi and anti-GST monoclonal antibodies (α GST). The positions of markers to the left of the panel depict protein standard molecular masses in kilodaltons. (TIF) [file ppat.1003567.s002.tif]
